# Syndecan-4 Is an Independent Predictor of All-Cause as Well as Cardiovascular Mortality in Hemodialysis Patients

**DOI:** 10.1371/journal.pone.0163532

**Published:** 2016-09-29

**Authors:** Andrzej J. Jaroszyński, Anna Jaroszyńska, Stanisław Przywara, Tomasz Zaborowski, Andrzej Książek, Wojciech Dąbrowski

**Affiliations:** 1 Institute of Medical Sciences, Jan Kochanowski University in Kielce, Kielce, Poland; 2 Department of Family Medicine, Medical University of Lublin, Lublin, Poland; 3 Department of Cardiology, Medical University of Lublin, Lublin, Poland; 4 Department of Vascular Surgery, Medical University of Lublin, Lublin, Poland; 5 Department of Nephrology, Medical University of Lublin, Lublin, Poland; 6 Department of Anesthesiology and Intensive Care, Medical University of Lublin, Lublin, Poland; University of Florida College of Medicine, UNITED STATES

## Abstract

**Background:**

Left ventricular hypertrophy is associated withincreased mortality in hemodialysis (HD) patients.Syndecan-4 plays a role in many processes that are involved in the heart fibrosis and hypertrophy.We designed this study to prospectively determine whether syndecan-4 was predictive of mortality in a group of HD patients.

**Methods:**

In total, 191 HD patients were included. Clinical, biochemical and echocardiographic parameters were recorded. HD patients were followed-up for 23.18 ± 4.02 months.

**Results:**

Syndecan-4 levels correlated strongly with geometrical echocardiographic parameters and ejection fraction. Relations with pressure-related parameters were weak and only marginally significant. Using the receiver operating characteristics the optimal cut-off points in predicting all-cause as well as cardiovascular (CV) mortality were evaluated and patients were divided into low and high syndecan-4 groups. A Kaplan–Meier analysis showed that the cumulative incidences of all-cause as well as CV mortality were higher in high serum syndecan-4 group compared with those with low serum syndecan-4 (p<0.001 in both cases).A multivariate Cox proportional hazards regression analysis revealed syndecan-4 concentration to be an independent and significant predictor of all-cause (hazard ratio, 2.99; confidence interval, 2.34 to 3.113; p<0.001)as well as CV mortality (hazard ratio, 2.81;confidence interval, 2.28to3.02; p<0.001).

**Conclusions:**

Serum syndecan-4 concentration reflects predominantly geometrical echocardiographic parameters. In HD patients serum syndecan-4 concentration is independently associated with all-cause as well as CV mortality.

## Introduction

Cardiovascular(CV) disease is the leading cause of mortality in hemodialysis (HD) patients. The CV mortality rate of HD patients is much higher than that of the general population, even after adjustment for age, race, gender, and comorbidities [1–3].Left ventricular hypertrophy (LVH) and cardiac fibrosis have been described as a frequent component of end-stage renal disease, and is observed in~70% of dialysis patients. In HD patients LVH is mainly attributed to hypertension, anemia, volume overload, arteriovenous fistulas, hyperparathyroidism, arterial stiffening, and oxidative stress [4–6]. It is well established that LVH is strongly associated with increased mortality in HD patients [5–7].

Syndecans are major cell-surface heparan sulfate proteoglycans that are detected at the surface of most human cells. It consists of an ectodomain carrying heparin sulfate- or chondroitin sulfate-rich glucosaminoglycan chains, a transmembrane domain, and a short cytoplasmic tail [8–12]. Syndecan-4 connects extracellular matrix proteins to the cardiomyocyte cytoskeleton and mediates signals transduction pathways activated by growth factors and cell surface receptors. It can also cooperate with other molecules, including extracellular matrix components, enzymes, and clotting factors. Syndecan-4 plays a role in many processes including inflammation, wound healing tissue regeneration, and angiogenesis[12–17]. It has been demonstrated that syndecan-4 may play a role in collagen cross-linking, left ventricle stiffness, and cardiac fibrosis[9, 15, 16, 18], and is involved in cardiac injury and repair, including granulation tissue formation, and neorevscularization after myocardial infarction [10, 11, 14, 19, 20]. Moreover, some recent clinical studies have demonstrated that syndecan-4 may be a useful biomarker for patients with cardiomyopathy [17, 21].

Given that syndecan-4 is the only syndecan with ubiquitous distribution (including heart and endothelial cells) [22–24], is essential for development of cardiac hypertrophy [15, 25], is required for endothelial alignment in flow, and is a potent antiatherosclerotic molecule[23], we hypothesized that it might be a potential biomarker useful in CV risk stratification in HD patients.

We designed this study to prospectively determine whether syndecan-4 was predictive of all-cause and CV mortality in a group of HD patients.

## Subjects and Methods

### Study population

The study included adult chronic HD patients treated at dialysis centers in Lublin (Poland). The exclusion criteria were: HD treatment less than 1 month, and advanced malignant diseases limiting the chance of a 3 months survival. All patients gave a written consent, and the studies were approved by members of the Ethical Committee of Medical University of Lublin.

### Biochemical measurements

The following biochemical parameters were measured by automated analyzers at the beginning of the evaluation: sodium, potassium, calcium, phosphorus, Ca×P product, creatinine, urea, hemoglobin, intact parathormone (PTH), C-reactive protein (CRP), total protein, albumin, total cholesterol, HDL cholesterol, triglycerides (TG), LDL cholesterol, and troponin T.Syndecan-4 as well as NT-proBNP were measured by the ELISA method (Biomedica, Poland).Serum syndecan-4 was also evaluated in 54 controls; the group’s gender distribution and age range were similar to the group of patients. Blood was obtained after at least 8 h fasting.

### Echocardiographic examination

An experienced cardiologist who was blinded to the clinical data of the study subjects performed the echocardiographic studies according to the recommendations of the American Society of Echocardiography [26–28]. Left ventricle ejection fraction was calculated using a Simpson`s method. The LVM was calculated according to the formula of Devereux and Reicheck [29] and this was indexed for body surface to obtain the LVMI. LVH was defined by an LVMI over 130 g/m^2^ in males or over 110 g/m^2^ in females. Ejection fraction < 50% was considered systolic dysfunction. All echocardiographic measurements were performed in the morning after dialysis [30].

### Follow-up data

All patients were followed for at least 36monthsfrom the baseline assessment or until death or renal transplant. All-cause mortality as well asCV mortality were used as endpoints. CV death was defined according to Standardized Definitions for End Point Events in Cardiovascular Trials [31]. All events were independently determined by two physicians. In the case of divergent opinions, the event was verified by an expert in cardiology.

### Statistical analysis

Statistical analysis was carried out as described in detail previously [32]. Results were tested for normality by using Kolmogorov–Smirnov test. When normally distributed, continuous variables were expressed asmean ± SD, and as median and range when non-normally distributed. Categorical data were expressed as frequencies and percentages. Linear regression analysis was assessed using the Pearson correlation coefficient. When the distribution pattern did not match with normal distribution, data were converted by logarithmic transformation and then statistic tests were performed. The receiver operating characteristics curves (ROC) was to determine optimal cut-off points in predicting all-cause as well as CV mortality. The optimal cut-off value for syndecan-4was defined as the value on the ROC curve that was associated with the minimum Euclidean distance from the curve to the upper left corner of the graph, using the following formula: (1 –sensitivity)^2^ + (1 –specificity)^2^ [33].For the survival analysis, patients were divided into groups according to the cut-off syndecan-4 level determined by the ROC analysis. Survival was measured beginning from the day of baseline examination until death or censoring. If patients underwent renal transplantation they were censored on the day of their last dialysis. Cumulative survival curves were constructed by using the Kaplan–Meier method for all-cause mortality andCV mortality. Differences between patient groups were assessed by use of the log-rank test. Relationships between baseline parameters and endpoints were analyzed with Cox proportional hazard regression analysis. In the univariate analyses, parameters that showed differences between high and low syndecan-4 groups were entered. Explanatory variables with a p value<0.15 in the univariate analysis were entered into a multivariate analysis. Probability values of p<0.05 were accepted as significant.

## Results

Of the total of 202 HD patients initially identified, 7 patients were excluded due to advanced malignant diseases limiting the chance of a 3months survival(4 patients) or treatment less than 1 month (3 patients). The remaining195 HD patients (105 females and 90 males), aged 38–86 years (mean 69.6 ± 7.93), who remained on HD from 1 to 147 months (mean 46.33 ± 25.63) entered the study. The causes of end-stage renal disease (ESRD) were diabetes (n = 85), chronic glomerulonephritis (n = 40), hypertensive nephropathy (n = 21), polycystic kidney disease (n = 7), obstructive nephropathy (n = 6), chronic pyelonephritis (n = 4), and unknown/unsure (n = 32). Baseline characteristics for enrolled patients are shown in Table 1. Out of 195 patients who qualified for the study, 79.5% were taking angiotensin-converting enzyme inhibitors/angiotensin receptor blockers, 85.6% beta-blockers, and 60.5% statin. A history of myocardial infarction (MI) was present in 25.1% of patients and 52.8% were diabetic. Hypertension was observed in 85.1% of patients. LVH was found in 66.1% of patients, systolic dysfunction was found in 24.1%.Serum syndecan-4 was higher in patients compared with controls (15.9 ± 9.78 and 5.04 ± 2.08 respectively, p<0.001).

**Table 1:** Baseline characteristics of patients.

parameter	All patientsn = 195	Low syndecan-4 n = 126	High syndecan-4 n = 69	p
Age (years)	69.5 ± 7.95	66.7 ± 7.08	74.6 ± 6.35	< 0.001
HD vintage (months)	46.2 ± 25.74	45.7 ± 24.78	47.1 ± 23.64	0.672
MI (%)	25.6	21.0	34.3	< 0.001
Diabetes mellitus (%)	57.1	54.8	61.2	0.012
Hypertension (%)	85.3	86.3	83.6	0.341
Smoking	13.1	13.7	11.9	0.493
Beta-blockers (%)	86.4	83.1	92.5	0.001
ACE/ARB (%)	75.4	77.4	71.6	0.285
Statins (%)	59.2	60.5	56.7	0.393
E/e`(n)	14.82 ± 5.28	13.96± 5.13	16.39 ± 4.77	0.011
LVMI (g/m^2^)	144.2 ± 42.74	131.2 ± 41.83	169.3 ± 31.68	< 0.001
EF (%)	56.83 ± 6.33	54.72 ± 6.01	60.72 ± 5.76	0.001
LAVI (ml/m^2^)	36.75 ± 8.24	35.24 ±7.24	38.34±7.75	0.012
Hemoglobin (g/dL)	11.07 ± 1.21	11.03 ± 0.98	11.21 ±1.22	0.590
Total cholesterol (mg/dL)	187.4 ± 37.35	188.1 ± 37.11	186.0 ± 35.87	0.674
LDL cholesterol (mg/dL)	116.9 ± 31.03	116.1 ± 30.13	118.3 ± 28.02	0.632
HDL cholesterol (mg/dL)	43.12 ± 18.11	43.4 ± 17.86	42.67 ± 14.45	0.711
Triglycerides (mg/dL)	172.1 ± 61.24	170.2 ± 59.54	175.7 ± 54.9	0.314
PTH, range (pg/mL)	394 (0.0–1212)	375 (0.0–924)	468 (0.0–1212)	0.215
Albumin (g/dL)	3.68 ± 0.37	3.69 ± 0.36	3.61 ± 0.32	0.775
CRP, range (mg/dL)	7.98 (0.22–112.1)	5.34 (0.22–67.4)	9.72 (1.18–112.1)	0.132
Troponin T, range (μg/L)	0.051 (0.00–0.775)	0.034(0.00–0.379)	0.098(0.032–0.775)	0.021
NT-proBNP (fmol/ml)	301.3 ± 135.4	276.7± 125.9	346.9 ± 101.8	0.096
Sodium (mmol/L)	137.8 ± 2.63	137.7 ± 2.61	138.0 ± 2.69	0.874
Potassium (mmol/L)	5.73 ± 0.72	5.69 ± 0.74	5.82 ± 0.63	0.642
Calcium (mmol/L)	2.48 ± 0.25	2.49 ± 0.25	2.47 ± 0.25	0.612
Phosphate (mmol/L)	2.24 ± 0.39	2.21 ± 0.23	2.30 ± 0.29	0.102
Ca x P product mg^2^/dl^2^	48.51 ± 10.62	46.9 ± 9.69	51.33 ± 9.87	0.024

CAD–coronary artery disease; MI–history of myocardial infarction; ACE/ARB—angiotensin-converting enzyme inhibitors/angiotensin receptor blockers;LVEDd–left ventricular end-diastolic diameter; LVESd–left ventricle endsystolic diameter; PWDd–posterior wall diastolic diameter; PWSd–posterior wall systolic diameter; IVSDd–interventricular septum enddiastolic diameter; IVSSd–Interventricular septum endsystolic diameter; E/e`—early transmitral flow velocity / early diastolic velocity of the mitral valve annulus;LVM–Left ventricular mass;LVMI–Left ventricular mass index; LVH–Left ventricular hypertrophy; EF–Ejection fraction; LAVI–Left atrial volume index; PTH–parathormon; CRP–C-reactive protein; NT-proBNP—N-terminal pro-hormone brain natriuretic peptide.

The results of univariate linear regression analysis showed significant, however weak correlation between serum syndecan-4 and NT-proBNP concentrations. The relation between syndecan-4 and troponin T was not significant. Similarly, there was no dependence of syndecan-4 on gender or age. With regard to echocardiographic parameters syndecan-4 levels correlated with geometrical parameters (LVMI, LVEDd, IVSd, PWd), ejection fraction, as well as pressure-related parameters (E/e`, LAVI). It should be noted, however, that relations with pressure-related parameters were weak and only marginally significant. Relationship between serum syndecan-4 and biochemical as well echocardiographic parameters are depicted in Table 2.

**Table 2:** Associations between syndecan-4 and echocardiographic as well as biochemical parameters.

	parameter	r	p
Syndecan-4	Age	0.121	0.109
	HD vintage	0.073	0.312
	EF	- 0.531	<0.001
	LVEDd	0.446	<0.001
	IVSDd	0.371	<0.001
	PWDd	0.332	<0.001
	LVMI	0.421	<0.001
	E/e`	0.208	0.011
	LAVI	0.199	0.014
	NT-proBNP	0.201	0.013
	Troponin T	0.075	0.324

LVEDd–left ventricular end-diastolic diameter; IVSDd–interventricular septum enddiastolic diameter; PWDd–posterior wall diastolic diameter; LVMI–left ventricle mass index; E/e`—early transmitral flow velocity / early diastolic velocity of the mitral valve annulus; LAVI–left atrial volume index

The ROC characteristics curves were created to determine optimal cutoff points in predicting all-cause as well as CV mortality. With regard to all-cause mortality the value for (1 –sensitivity)^2^ + (1 –specificity)^2^ was minimal when the serum syndecan-4 level was 22.6 ng/ml (sensitivity of 71.4% and specificity of 84.1%). In the case of CV mortality the optimal cut-off point was 21.9ng/ml (sensitivity of 70.4% and specificity of 85.3%). In 68 patients (34.9%) serum syndecan-4 exceeded 22.6 ng/ml, and in 69 (35.5%) patients syndecan-4 was above 21.9 ng/ml. Given that optimal cut-off values for both all-cause and CV mortality were very similar, for further analysis the lower level was assumed. On the basis of optimal cut-off value patients were divided into low and high serum syndecan-4 groups.

High syndecan-4 patients were older (p<0.001),more often had a history of MI (p < 0.001), had higher prevalence of diabetes mellitus (p = 0.012), and were more likely to be on beta blocker therapy (p = 0.001). With regard to echocardiographic parameters, high syndecan-4 patients had higher LVMI (p<0.001),LAVI and E/e`values (p = 0.012 and p = 0.011, respectively)and lower EF (p < 0.001) in comparison to patients with low syndecan-4 patients. With regard to biochemical parameters, high syndecan-4 patients had higher troponin T level (p = 0.021) as well as Ca x P product (p = 0.024).

Over the mean follow-up period of 25.08 ± 4.32 months (range 1–36 months), there were 76all-cause deaths (39.0%), and the mortality rate was 13% per year. CV deaths accounted for 52.6% of all death. Eleven patients were transplanted. The incidence of all-cause deaths was as follows: in high syndecan-4group 58.1% and in low syndecan-4 group 27.8% (p<0.001). The incidence of CV mortality was as follows: in high syndecan-4 group 40.1% and in low syndecan-4 group 17.9% (p<0.001). All but one transplanted patients came from the high syndecan-4 group.

**Fig 1:**
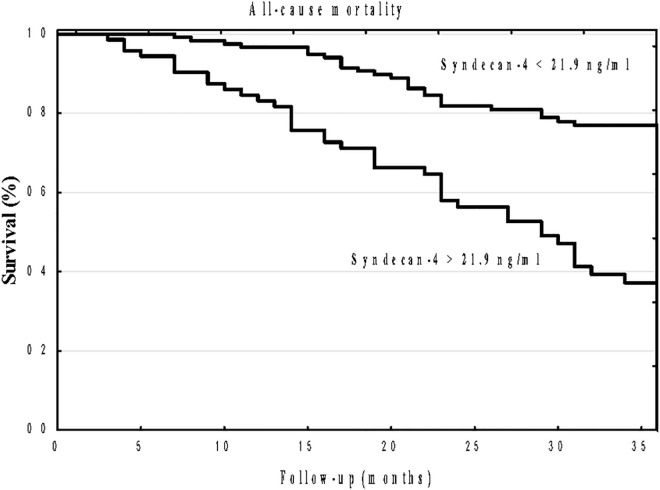
Kaplan–Meier survival plots for all-causer mortality in hemodialysis patients stratified by serum syndecan-4 optimal cut-off value p<0.001

**Fig 2:**
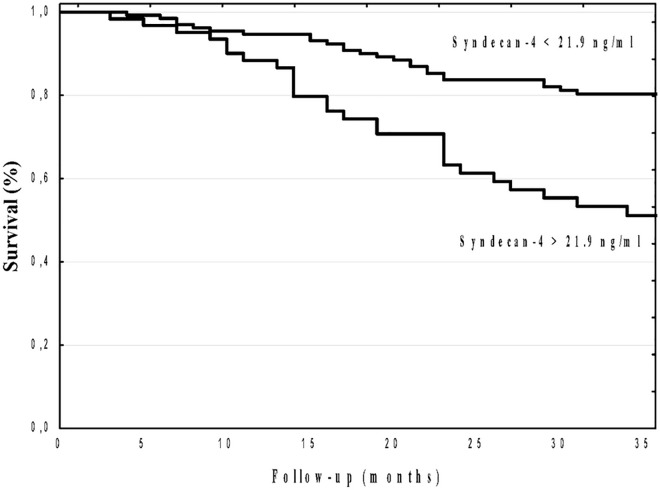
Kaplan–Meier survival plots for cardiovascular mortality in hemodialysis patients stratified by serum syndecan-4 optimal cut-off value p<0.001

A Kaplan–Meier analysis showed that the cumulative incidence of all-cause mortality was significantly higher in high syndecan-4 patients compared to low syndecan-4 group (log-rank, p<0.001; Fig 1). Similarly, a Kaplan–Meier analysis showed that the cumulative incidence of CV mortality was significantly higher in high syndecan-4 patients compared to low syndecan-4 group (log-rank, p<0.001; Fig 2).

To control for possible confounders, multivariate analysis was performed using a model consisting of univariate predictors of cardiac mortality. The results of the univariate and multivariate Cox proportional hazard regression analyses are shown in Tables 3 and 4. A multivariate analysis selected age [hazard ratio (HR) 1.57, p<0.001], syndecan-4 [HR 2.99, p = <0.001], and troponin T [HR 1.55 p = 0.025] as well as age [HR 1.70, p<0.001], syndecan-4 [HR 2.81, p<0.001], and LAVI [HR 2.28, p = 0.011],as independent predictors of all-cause and CV mortality, respectively.

**Table 3:** Uni- and multivariate predictors of all-cause mortality.

Parameter	Univariate HR(95% CI)	p	Multivariate HR(95% CI)	p
Age	1.98 (1.46–2.41)	<0.001	1.57 (1.18–1.91)	<0.001
Diabetes mellitus	1.45 (0.89–1.87)	0.009	1.12 (0.47–1.75)	0.347
History of MI	1.42 (0.92–1.74)	0.005	1.39 (0.71–2.32)	0.178
Beta-blockers	0.95 (0.51–2.32)	0.218		
Calcium x phosphate product	1.61 (0.84–3.22)	0.511		
LVMI	1.28 (0.66–2.25)	0.179		
EF	2.21 (1.65–3.23)	0.087	1.83 (0.82–3.42)	0.372
E/e`	2.49 (1.74–3.11)	0.011	1.78 (0.91–3.06)	0.245
LAVI	1.53 (1.12–2.14)	0.103	1.21 (0.75–2.57)	0.268
Troponin T	2.13 (1.79–2.65)	<0.001	1.55 (0.94–1.93)	0.025
Syndecan-4	2.94 (2.39–3.56)	<0.001	2.99 (2.34–3.11)	<0.001

HR, hazard ratio; CI, confidence interval; MI, myocardial infarction; LVMI, left ventricular mass index; EF, ejection fraction; E/e`—early transmitral flow velocity / early diastolic velocity of the mitral valve annulus; LAVI–left atrial volume index.In the multivariate analyses, parameters with a p≤ 0.15 were entered.

**Table 4:** Uni- and multivariate predictors of cardiovascular mortality.

Parameter	Univariate HR(95% CI)	p	Multivariate HR(95% CI)	p
Age	1.12 (0.99–1.43)	<0.001	1.72 (0.91–1.62)	<0.001
Diabetes mellitus	0.92 (0.41–1.97)	0.213		
History of MI	1.42 (0.92–1.74)	0.005	1.39 (0.71–2.32)	0.178
Beta-blockers	1.04 (0.31–2.65)	0.348		
Calcium x phosphate product	1.51 (0.44–3.32)	0.547		
LVMI	2.98 (2.34–3.37)	0.005	2.25 (1.42–3.49)	0.138
EF	3.41 (1.85–5.13)	0.217		
E/e`	3.34 (2.16–4.21)	0.107	2.72 (1.44–3.75)	0.134
LAVI	2.93 (2.19–3.53)	0.002	2.28 (1.43–2.88)	0.011
Troponin T	1.78 (1.09–2.54)	0.023	1.17 (0.59–2.31)	0.112
Syndecan-4	3.43 (2.72–4.06)	0.001	2.81 (2.28–3.02)	<0.001

HR, hazard ratio; CI, confidence interval; MI, myocardial infarction; LVMI, left ventricular mass index; EF, ejection fraction. In the multivariate analyses, parameters with a p≤ 0.01 were entered.

## Discussion

Our study generated three major findings: (i) serum syndecan-4 concentration is increased in HD patients compared with controls, (ii) serum syndecan-4 concentration correlated predominantly with geometrical echocardiographic parameters, (iii) serum syndecan-4 level is an independent predictor of both all-cause and CV mortality in HD patients.

Results of the present study have revealed that syndecan-4 level was higher in HD patients than in controls. It can result from the fact that LVH is prevalent in HD patients, and syndecan-4 is increased in proportion to the LVMI. The prevalence of LVH in our study (64.4%) was comparable to the prevalence of LVH reported by other authors [5, 7]. The other reason may be the accumulation of syndecan-4 as a result of kidney failure, however, according to our knowledge, the effect of renal function and influence of HD process on syndecan-4 metabolism remains unknown. It is difficult, however, to compare directly our results to the available data from other populations. This is largely due to the fact that syndecan-4 is not measured using reference standards.

In our study syndecan-4 correlated significantly both with geometrical and pressure-related echocardiographic parameters, however, relations between syndecan-4 and pressure-related parameters were weak and only marginally significant. Additionally, syndecan-4 correlated with EF. The results of the current study are in agreement with the results of Takahashi et al. [17], who also found relations between syndecan-4 and geometrical echocardiographic parameters as well as EF. In contrast to our results Takahashi et al. [17] have not revealed significant relations between syndecan-4 and pressure-related echocardiographic parameters as well as NT-proBNP. This may be due to differences between the studied groups of patients regarding the causes of cardiac hypertrophy. Overhydration is an important factor contributing to LVH in HD patients, and fluid overload is associated with pressure-related echocardiographic parameters, such as LAVI as well as E/e`[34, 35].Syndecan-4 plays a role in a myriad of processes that are involved in the heart failure pathogenesis[12–17, 25, 36].Results of previous studies have demonstrated that syndecan-4 is essential for development of cardiac hypertrophy [9, 15, 25], and syndecan-4 production is increased in the repair, but not undamaged region after myocardial infarction [10]. Weak relation between syndecan-4 and NT-proBNP may result from the fact that both biomarkers may represent somewhat different aspects of failing heart.

To our knowledge, this is the first study that has demonstrated the prognostic utility of syndecan-4 for predicting all-cause as well as CV mortality in HD patients. The Kaplan–Meier curves began to separate relatively early and then continued to stay separated until the end of follow-up for both all-cause as well as CV mortality. Moreover, multivariate Cox analysis revealed that syndecan-4 remained independent predictor after controlling for possibly confounders. The prognostic utility of syndecan-4 for predicting all-cause as well as cardiac death in HD patients is in concordance with some previous studies that have documented that syndecan-4 is a useful biomarker for predicting cardiac death for patients with chronic heart failure [17] as well as for predicting adverse left ventricle remodeling in patients with dilated cardiomyopathy [21].The pathophysiological mechanism of the association between syndecan-4 and HD patients mortality remains unknown. Syndecan-4 has been reported to promote hypertrophic response after myocardial infarction [15], and its level is elevated after myocardial infarction with a peak concentration corresponding to the repair phase [10]. Moreover, syndecan-4 plays a role in wound healing and angiogenesis [13]. Myocardial ischemia is the major reason of LVH in HD patients [5–7]. Coronary artery disease affects most of HD patients [32], and moreover HD process per se can induce transient myocardial ischemia (myocardial stunning), that may lead to myocardial dysfunction [37, 38]. Given that in our study syndecan-4 correlated with geometrical echocardiographic parameters and LVH, we can hypothesized that elevated syndecan-4 may reflects the severity of defense mechanisms against ischemia induced myocardial damage as well as may atherosclerosis. Further studies are required to answer the question of whether syndecan-4 is merely a marker of poor prognosis that reflects the high prevalence of LVH, contributing to all-cause as well as CV mortality, or if it potentially identifies more distinct pathophysiological mechanisms that underlie increased mortality.

The present study has some important limitations. First, the numbers of patients in the study was relatively small. Nevertheless, the population was large enough to demonstrate a predictive value for syndecan-4. Second, it is possible that serial rather than single measurements of syndecan-4 may have changed the results, making syndecan-4 either more or less useful in predicting clinical events in HD patients. Third, the effect of renal function as well an HD process on syndecan-4 concentration remains unknown.

## Conclusions

Serum syndecan-4 concentration reflect predominantly geometrical echocardiographic parameters. In HD patients serum syndecan-4 concentrationis independently associated with all-cause as well as CV mortality.
